# Ferdinand Magellan (1480–1521) – 500 years from the expedition: the first step towards globalization

**DOI:** 10.25122/jml-2022-1006

**Published:** 2022-05

**Authors:** Alexandru Vlad Ciurea, Mihai-Stelian Moreanu, Aurel Mohan, Dan Bentia

**Affiliations:** 1. Honorary Member of Romanian Academy; 2. Neurosurgery Department, Carol Davila University of Medicine and Pharmacy, Bucharest, Romania; 3. Neurosurgery Department, Sanador Clinical Hospital, Bucharest, Romania; 4. Neurosurgery Department, Faculty of Medicine, University of Oradea, Oradea, Romania; 5. Neurosurgery Department, Oradea County Emergency Clinical Hospital, Oradea, Romania

## INTRODUCTION

Globalization is the process by which ideas, knowledge, information, goods, and services are disseminated worldwide. Depending on the activity domain, globalization may encompass various aspects. In economics, globalization is directly linked to free trade and the free flow of capital between countries [1]. In terms of partnerships, globalization promotes the interactions and integrations between countries harboring the same interests. The idea behind economic globalization is that specialized countries could produce materials and products more efficiently, with high benefits within a very short timeframe. Low-priced materials could be accessed easily and sold in high-consumer demand markets.

Another facet of globalization is telecommunications and the internet [2]. Access to information and its flow has increased in the past few decades. With the emergent power of 5G communication, the process is several times faster than in past times. This linkage between countries has moved the interaction between people to an altogether different level. Nevertheless, this pandemic showed us that there are still many opportunities to be found by switching many of our daily meetings to the online environment.

Therefore, globalization is the main trend typical of the 21^st^ century. However, what course has this concept followed? History has shown us that this process has been happening for hundreds of years, at least in some way.

The most prominent empires in the history of humankind, such as the Greek or Roman Empires, united different cultural and political systems under one governmental system that rules over vast territories. They established their own economic, cultural and political system [3].

The Silk Road trade was one of the most outstanding commercial routes in the history of Asia from 130 B.C. to 1453 A.D. Merchants, goods, and travelers have made their way from China through Central Asia and the Middle East to Europe.

This year marks the 500^th^ anniversary of the first expedition around the Earth initiated and coordinated by Ferdinand Magellan, the greatest explorer of the Age of Discovery. The journey was an otherworldly adventure that opened new scientific horizons for humanity. This expedition was a confirmation of what Eratosthenes of Cyrene said in Ancient times, who argued that the Earth is round.

Magellan (Fernão de Magalhães) was born in 1480, in the city of Sabrosa in northern Portugal, to a family of original patricians. At the age of 12 he was sent to the Royal House of Portugal where, along with his brother, was lifted as a page and received a good education that included cartography, mathematics, astronomy, and navigation [4].

**Figure 1:**
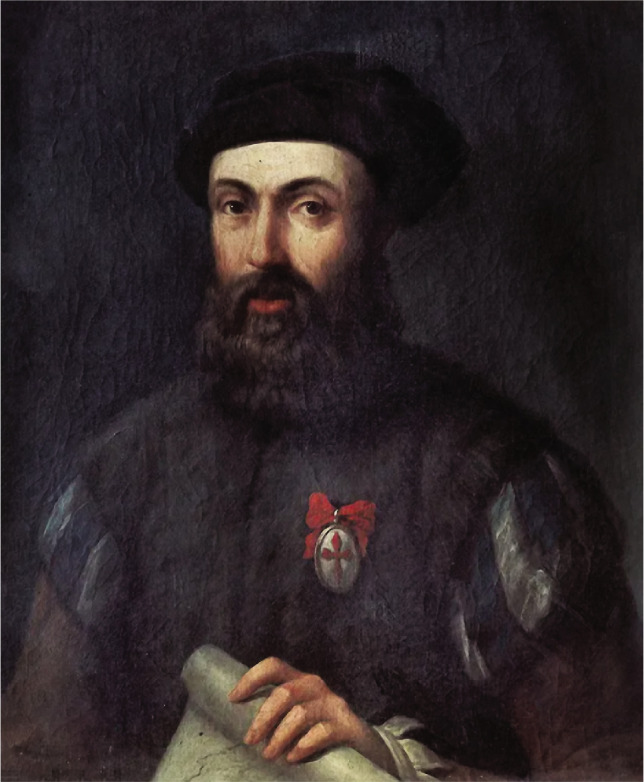
Portrait of Ferdinand Magellan dating from 1848, a copy after an engraving from 1784 illustrating the circumnavigation of 1521.

At the age of 25, in 1505, Magellan was enrolled in the Portuguese army and made multitudinous passages along the beachfronts of Africa and the East for nearly two decades, taking part in naval battles, first as a soldier and later on as an officer. During these adventures, Magellan achieved elevation for his zeal in battles and his organizational abilities (Figure 1).

He was injured in Morocco, which rendered him lame for the rest of his life. He also had disagreements with the Portuguese rulers in Morocco and later fell out of favor with King Manuel I.

Even in these trying circumstances, he remained undeterred and planned an expedition to the Moluccas (Spice Islands) by trekking west through South America with the support of his friend, cosmographer Rui Faleiro.

Seeing that his projects were unlikely to be accepted in Portugal, Magellan went to Spain in 1517, settling in Seville with the support of the Portuguese Diego Barbosa, and was received by the Court of Spain to present to King Charles I his proposal to reach the Spice Islands by journeying westward. The concept was approved, and the Spanish government agreed to fund it. Magellan was chosen Commander, with the consent of the King and the “Council of the Indies” and began planning the journey in 1518 [5].

**Figure 2:**
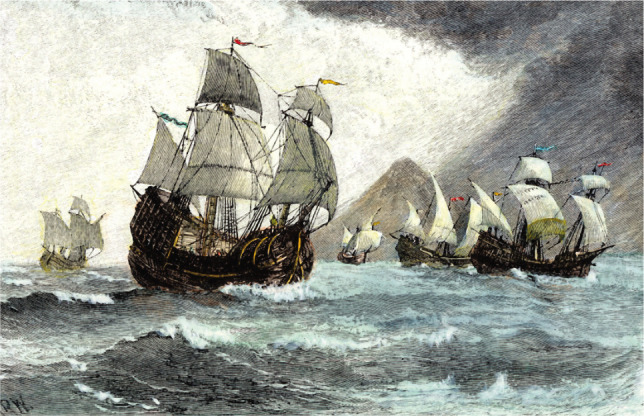
Portuguese explorer Ferdinand Magellan's fleet of five ships after their departure from Spain on September 20, 1519; wood engraving, 19^th^ Century.

The expedition consisted of 5 armed ships packed for the discovery of a new spice route (the Trinidad, Magellan's flagship, and the ships San Antonio, Concepción, Victoria, and Santiago) with a crew of 270 persons, predominantly Spaniards but also Portuguese, Italians, French, Germans, Greeks, and others (Figure 2).

On September 20, 1519, the Atlantic voyage began from the Andalusian port of Sanlucar de Barrameda, the departure point for Christopher Columbus' first mission, who discovered America in 1492. They arrived in Santa Lucia Bay (now Rio de Janeiro) on November 13, 1519, after a 2-month cruise and a brief stay in the Canary Islands. They traveled south to San Julián Bay after exploring the La Plata River (Silver River) estuary, where Magellan, who was well-versed in the southern hemisphere, decided to stop and winter [6].

**Figure 3:**
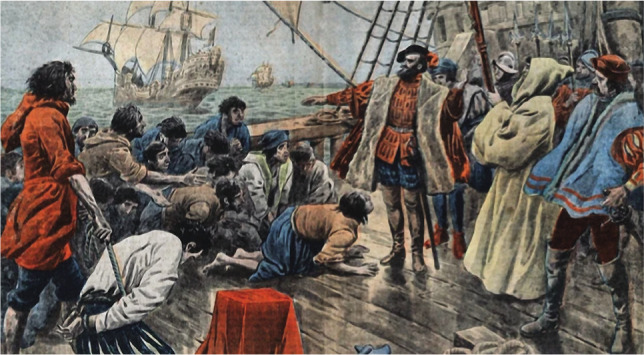
The crew pledge their allegiance to Magellan after an unsuccessful mutiny. For many of them it would be a hollow oath. © Photo by Stefano Bianchetti/Corbis via Getty Images.

This was also the last point that previous explorers could reach. Fearful of the unknown and dissatisfied that the expedition was being directed by a Portuguese man, the Spanish commanders aboard San Antonio, Concepción, and Victoria, led by Juan de Cartagena, plotted a rebellion, including some of the crew. Magellan put down the mutiny firmly and mercilessly but with considerable discretion and had the commanders of the three ships executed, with the exception of the insurrection's originator, who was instead marooned (Figure 3).

The expedition's continuance down the South American shores was marked by several uncertainties and harsh difficulties. The crew of the Santiago ship was rescued and distributed among the other ships after the ship was lost in a storm. Despite the callous conditions and lack of food, which included strong storms and worker dissatisfaction, Magellan continued to pursue the mission.

The opening to a strait, which would prove to be the most sought-after route between the Atlantic and Pacific Oceans, was discovered on October 21, 1520. Two ships were dispatched to conduct explorations of the strait, but one of them, the San Antonio, deserted and returned to Spain, crossing the Atlantic Ocean in the process.

The Strait of Magellan, originally called the *Canal de Todos los Santos*, is about 570 km long and separates the southern tip of South America from the Tierra del Fuego. Crossing the strait took 27 days and was very difficult due to rough underwater terrain, many rocks, strong sea currents, and frequent storms [6].

On November 28, 1521, the team of the 3 ships came to see the bright and calm area of the largest ocean on Earth, called by Magellan the Pacific Ocean (Mar Pacífico). The ocean was first seen 8 years before and named the South Sea (Mar del Sur) by the Spanish conquistador Vasco Balboa.

In an examination of the optimal crossing position concerning the wind dominating the Pacific Ocean, Magellan first sailed North along the West seacoast of South America (now Chile), and a month later, on December 18, 1521, the vessels sailed West and Northwest.

This inconceivable trip into the unknown unfolded over 3 months full of failings. The first areas encountered were most probably the island of Guam (ceded by Spain to the United States in 1899) and the archipelago of the Mariana Islands. In March 1521, a number of islands were discovered in the San Lazarus Archipelago (the Philippine Archipelago), the original population being largely converted to Christianity [7].

**Figure 4:**
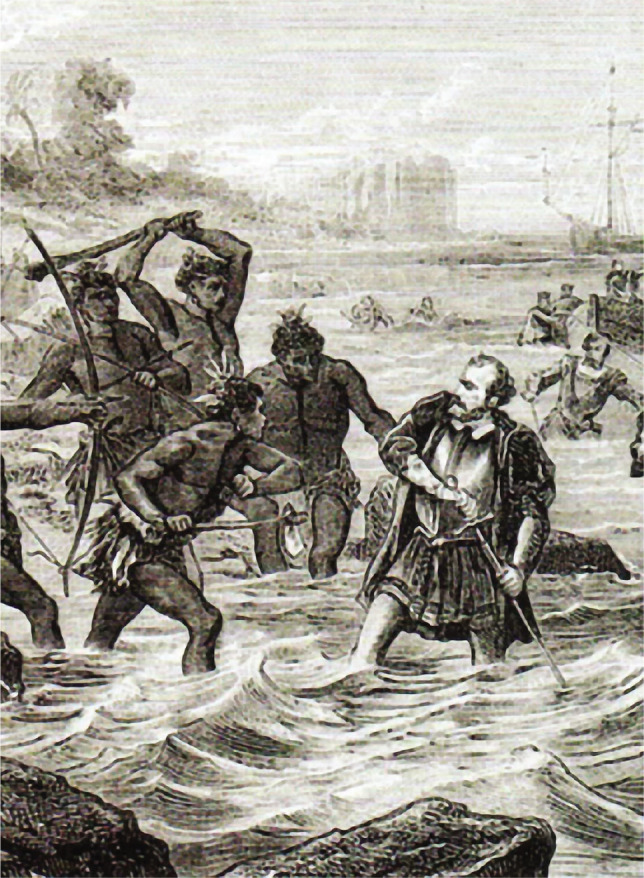
An illustration depicting the death of the Portuguese Navigator Ferdinand Magellan. Magellan was killed on April 27, 1521 during the Battle of Mactan on the Island of Cebu (present-day Philippines).

Ferdinand Magellan died on April 27, 1521, in a fight with the natives of Mactan Island near the port of Cebu (present-day Philippines) (Figure 4).

**Figure 5:**
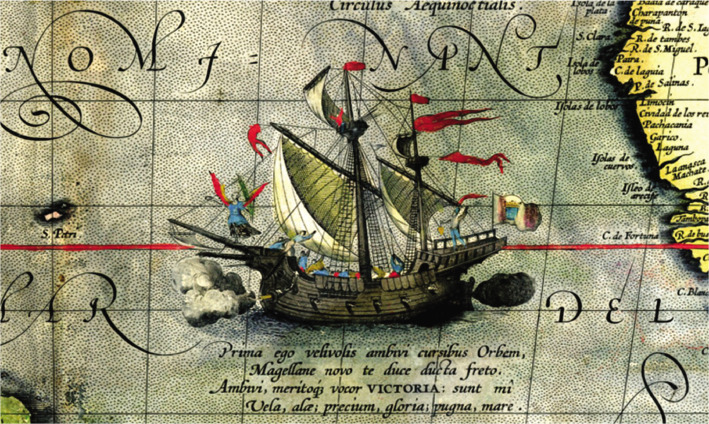
Ferdinand Magellan's ship the *Victoria*, detail from a map of the Pacific Ocean in Abraham Ortelius's *Theatro d'el orbe de la Tierra*, 1612.

The journey to the Moluccas (Spice Islands) was continued by the ships *Victoria* and *Trinidad*, which had to be abandoned for repairs (Figure 5).

Under the command of the Spaniard Juan Sebastián Elcano, the only boat to end its voyage back to Spain on September 6, 1521, was the *Victoria*, with only 18 persons on board (of the 270 who set out on the expedition 3 years previous) [8]. Magellan was considered a traitor by King Manuel I of Portugal because he never returned to his country [9].

Italian Antonio Pigafetta, chronicler and historian, was among the 18 sailors who returned to Seville and who reported and later published most of the data on the expedition, and Francisco Alba who handled the logbooks of some of the vessels he sailed on [10].

## CONCLUSIONS

Magellan heralded a new age of globalization regarding the knowledge and use of Earth's resources. Magellan's journey proved that the planet we live on has a spherical shape whose surface is dominated by the vast expanse of the oceans. By discovering the strait that bears his name and crossing the Pacific Ocean, Magellan proved the existence of the Planetary Ocean and became one of the forerunners of the knowledge of the latitudinal climatic differentiation to which the development of sea currents, violent storms, and winds is linked.

At the same time, due to his heroic actions, perseverance, and intelligence, which he employed in tackling the most unexpected dangers, Magellan can be ranked among the brilliant figureheads who broke new and important ground for the understanding and development of man's ability to face innumerable unforeseeable perils.

Magellan's journey was likened to man's first flight into space, as well as to the Moon landing that ushered in the “Space Age”.

The US Space Agency NASA paid tribute to the great discoverer by naming in his honor the first spaceship to explore the Solar System, which was launched with the help of the Atlantis spacecraft, “Magellan – NASA Solar System Exploration.”
